# Parental investment in child health in sub-Saharan Africa: a cross-national study of health-seeking behaviour

**DOI:** 10.1098/rsos.150460

**Published:** 2016-02-24

**Authors:** Caroline Uggla, Ruth Mace

**Affiliations:** Department of Anthropology, University College London, 14 Taviton Street, London WC1H 0BW, UK

**Keywords:** child health, parental investment theory, Demographic and Health Survey, health-seeking behaviour, sub-Saharan Africa, multi-level model

## Abstract

Parents face trade-offs between investing in child health and other fitness enhancing activities. In humans, parental investment theory has mostly been examined through the analysis of differential child outcomes, with less emphasis on the actions parents take to further a particular offspring’s condition. Here, we make use of household data on health-seeking for children in a high mortality context where such behaviours are crucial for offspring survival. Using Demographic and Health Survey (DHS) data from 17 sub-Saharan African countries, we examine whether maternal factors (age, health, marital status) and child factors (birth order, health, sex, age) independently influence parental investment in health-seeking behaviours: two preventative behaviours (malaria net use and immunization) and two curative ones (treating fever and diarrhoea). Results indicate that children with lower birth order, older mothers and mothers with better health status have higher odds of investment. The effects of a child’s sex and health status and whether the mother is polygynously married vary depending on the type of health-seeking behaviour (preventative versus curative). We discuss how these results square with predictions from parental investment theory pertaining to the state of mothers and children, and reflect on some potential mechanisms and directions for future research.

## 1. Introduction

A child is highly dependent on its parents when in need of healthcare. When parents do not take action to prevent or cure illnesses that pose a threat to child survival, it is often considered to be the result of constraints, such as lack of resources or lack of knowledge. Indeed, population health scientists examining parental health-seeking behaviours have clearly demonstrated that higher maternal education and wealth, and better access to health services are associated with higher levels of health investment in children [1–4]. However, considerable variation in parental health-seeking exists even between households with comparable levels of education, wealth and healthcare access [5–7]. This implies that other factors, such as characteristics of the parents and children, can help explain investment biases within and between households. Evolutionary models provide one framework with which to predict how much a parent should invest either in a particular offspring or in alternative fitness enhancing functions. Evolutionary parental investment theory posits that parents will act in a manner that maximizes their own inclusive fitness, i.e. genetic representation in future generations. Investment decisions must be weighed up against potential allocations of investment to alternative dimensions of fitness, such as investment in alternative offspring or the embodied capital of the parent. Thus, parental investment theory highlights that the health of a particular offspring—although important for the offspring’s fitness—is not the only priority of the parent.

Parental investment can be defined as any action by a parent that benefits the offspring but incurs a cost to the parent’s ability to invest in other offspring [8] or more broadly as investment that incurs costs to any other fitness enhancing activities [9]. Conflicts between parents and offspring result when the share of resources that it is optimal for the offspring to extract is higher than the share that is optimal for the parents to give up; parents might maximize their own fitness by preserving energy for their own somatic health, mating effort or other offspring [10]. So when should parents favour investment in a particular offspring over alternative strategies? Put broadly, factors that are associated with higher reproductive value, i.e. higher expected genetic contribution to future generations [11], of an offspring should increase the payoffs parents can expect from health investment in that offspring. The particular characteristics that make an individual more competitive as a mate, or those that are likely to result in higher reproductive success for some other reason, will depend on a specific set of assumptions; an offspring’s birth order, sex and health as well as age and health of the mother are all factors that have been explored in terms of how they influence parental investment (see e.g. [12] for review).

Studies of human parental investment have often been carried out by evolutionary anthropologists who have collected rich data from a single field site over long periods of time (e.g. [13,14]). This method of research is often necessary to enable careful consideration of the local socioecological context that might shift fitness costs and benefits and in order to understand behavioural diversity between populations. But with this approach comes the drawback that many central evolutionary hypotheses are tested on modest sample sizes and studies may differ in the factors they adjust for [15]. Labour intensive data collection means that cross-cultural comparison is often not feasible, and perhaps not desirable, if there are vital differences in data collection methods between studies. Consequently, while we have many sophisticated and rigorous accounts from diverse and marginalized populations, there is less evidence of the effects of key factors on parental investment based on broad and comparable cross-country data.

Evolutionary anthropologists and others have often used child outcomes such as birth weight, height-for-weight, illnesses or age-specific survival as approximations of investment made by parents [12,16]. Data on actual parental behaviour are generally more onerous to collect than the outcomes that are presumed to result from parental behaviour. While parental investment and child outcomes might overlap, relying on child outcomes as a proxy for parental investment might obscure effects due to other factors that influence child health, parental behaviour and the trade-offs between them. For example, parents with higher levels of resources or those who live in environments with lower disease burden are more likely to have offspring in good health without having to pay costs to achieve such outcomes. There might be many reasons why, for instance, children with higher birth order could have worse health outcomes than their older siblings, and parental bias in allocation of resources is only one potential pathway. In this study, we aim to offer a complement to studies that contribute with detailed examinations of parental investment dynamics in small-scale populations, by drawing on the breadth of cross-country data and by conceptualizing health-seeking behaviour as a form of parental investment.

We use household surveys from sub-Saharan Africa (SSA), where the most common causes of child death are preventable diseases that could be mitigated by deploying health technologies such as malaria bed nets and immunization and by prompt treatment of fever and diarrhoea. Our aim is to test how various key factors that might be linked to the reproductive value of the child and of the mother are associated with these four different health-seeking behaviours. Scholars trying to understand why health-seeking behaviour is not more widespread often focus on different sets of determinants, or come from experts of a particular disease, e.g. malaria, who consider behaviour relevant to that health issue, thereby impeding understanding of whether any observed effects are generalizable to other health-seeking behaviours. From a parental investment perspective, we expect that a higher reproductive value of a child should correspond to higher levels of investment in any given health-related behaviour. Nevertheless, as we have data on both preventative and curative health-seeking behaviours, we compare determinants of behaviours that might only generate payoffs in the future (preventative health-seeking) with those that aim to avert or ameliorate health dangers already incurred (curative health-seeking). In the discussion, we consider how our results square with sometimes competing hypotheses outlined from an evolutionary parental investment framework.

## 2. Data and methods

### 2.1 Data

The Demographic and Health Surveys (DHSs) are nationwide household surveys (www.measuredhs.com) and a gold standard resource of child and maternal health in developing countries. Our sample consists of data from mother–child pairs (*n*=9841–42 096, depending on the health-seeking behaviour) from 17 SSA countries (Burkina Faso, Cameroon, Congo (DR), Ethiopia, Ghana, Guinea, Lesotho, Liberia, Malawi, Mali, Niger, Rwanda, Senegal, Sierra Leone, Swaziland, Zambia and Zimbabwe) collected between 2003 and 2008. We use data on one child per woman, the latest born child (aged 0–59 months) as these health-seeking behaviours concern children under the age of 5 and data on multiple children per woman are scarce.

### 2.2 Measures of health-seeking behaviour

We use four different measures of health-seeking behaviour, reported by the mother: whether the child received (any) medication or treatment for fever or cough suffered in the 14 days preceding the survey (fever treatment), whether a child who had diarrhoea in the 14 days preceding the survey received oral rehydration solution (ORS), whether the child slept under a bed net on the night preceding the survey (bed net use) and whether the child had received full immunization coverage (defined as three doses of diphtheria–tetanus–pertussis (DTP), the Bacille Calmette–Guérin vaccine against tuberculosis, three doses of polio vaccine and one dose against measles). We cannot know for certain who took the decision to invest in a given child, but the mother is the most likely carer and the one who answers the survey questions on child health investment. These measures are good indicators of direct parental investment that aim to avoid child mortality and cover different aspects of health-seeking behaviour, in being preventative and curative, habitual (bed net use) and responses to events (fever treatment and ORS provisioning). They are specific to the focal child, except for bed net use that can benefit several children and the mother at the same time. Treatment for fever captures whether any type of treatment was sought, including both traditional and western remedies, as we are primarily interested in the actions and intentions of parents rather than the efficacy of a chosen treatment. (For mean level of health-seeking behaviour by country, see the electronic supplementary material, table S1). It is difficult to know the financial impact health-seeking behaviours have on a given household, as this might vary on a local level, however we include measures of possible barriers to healthcare (see below).

### 2.3 Independent variables

The independent maternal variables used are age (categorized in bands of 5 years, except the categories 40–44 and 45–49 years which were collapsed for sample size reasons), highest level of education (no formal education, primary, or secondary or higher), wealth, marital status (monogamous, polygynous or not married) and maternal health (weight-for-height, adjusted for pregnancy status). Poor maternal somatic condition might lead mothers to prioritize their own health over that of the offspring if there is a trade-off between maternal and child health allocations. Wealth is based on the DHS wealth index comprising a list of assets measured at household level and made into quintiles. Note that one important limitation of the DHS is that there is some variation between countries in how the household is defined, and this has impact on the household wealth measure [17]. The child variables are age, sex, number of older siblings and child health status, measured in terms of stunting (height-for-age) and wasting (weight-for-height) and categorized into normal/mild, moderate or severe stunting/wasting based on Z-scores in line with the standard WHO categorizations. To enable cross-country comparison, stunting and wasting in the DHS is defined as two standard deviations below the ‘international reference population’, which consists of US children who were selected because they were thought to be exposed to ‘optimal’ conditions [18]. This approach is based on the idea that all children have ‘similar genetic potential for growth’, but it is worth bearing in mind that it ignores the fact that being small might be disadvantageous in one context but advantageous in another. To address the issue of variation in access to health services, we include four community-level measures (over and above urban/rural residence): a subjective measure of distance as a problem for healthcare (the proportion of women in a cluster, primary sampling unit, who consider distance to health clinic a ‘big problem’ for seeking care for herself), a national measure of number of physicians per 100 000 population (from WHO), a logged HIV rate and an under-5 mortality rate (both on sub-national regional level) to adjust for differences in levels of disease pressures.

### 2.4 Statistical analysis

We run multilevel models where woman–child pairs (level 1) are clustered within 152 sub-national regions (level 2) within 17 countries (level 3). For the immunization model children younger than 12 months were excluded, because they were too young to have received the full immunization coverage, and for the bed net models three malaria-free sub-national regions were excluded. Covariates were added in blocks (model (a) only environmental controls, model (b) environmental controls + maternal variables, model (c) environmental controls + child variables and model (d) full model) in order to evaluate the impact of the variables on one another (see the electronic supplementary material, tables S2–S5). The full models are presented in the Result section. Analyses were performed using runmlwin in Stata 12. Second-order penalized quasi-likelihood followed by MCMC estimation was used to fit the models, and the model-fit statistic deviance information criterion (DIC) was used to compare models [19]. Number of physicians per 100 000 (country level) and logged HIV did not improve the DIC and were dropped.

## 3. Results

About one-fifth of the children under 5 slept under a bed net the night preceding the survey, and 35% and 39% were given ORS for diarrhoea and treatment for fever, respectively. Almost half of the children between 1 and 5 years had received full immunization coverage (table 1). The modal mother was aged between 25 and 29 years, had no formal education and resided in a rural area. Among the children, 22% were firstborns, and 35% and 12% were at least moderately stunted and wasted, respectively. Below we report on the results from the models on health investment; unless otherwise stated, the effects are consistent across the four outcomes.

**Table 1.** Distribution of heath seeking-behaviours by maternal, child and cluster characteristics.

	curative measures		preventative measures	
	fever treatment *n*=18 779	ORS *n*=9841	bed net use *n*=42 096	immunization *n*=32 027
*maternal*	%	%	%	%
age (years)				
15–19 (7%)	38	35	24	27
20–24 (24%)	38	37	25	36
25–29 (25%)	39	35	27	37
30–34 (19%)	40	33	25	38
35–39 (14%)	38	32	24	38
40–49 (11%)	34	33	20	41
education				
none (53%)	33	28	23	32
primary (31%)	41	44	24	40
secondary or >(16%)	50	47	30	47
wealth				
poorest (21%)	32	29	19	30
poor (21%)	34	33	23	33
middle (22%)	37	33	24	36
rich (20%)	42	38	26	39
richest (17%)	52	43	34	48
marital status				
not married (11%)	42	44	15	41
monogamous (63%)	39	36	27	37
polygynous (26%)	35	28	23	34
wasted				
normal–mild (59%)	41	37	26	38
moderate (32%)	35	32	23	36
severe (9%)	32	26	19	35
*child*				
sex				
girl (49%)	37	34	25	37
boy (51%)	40	36	25	37
age (months)				
0–11 (21%)	38	31	27	9
12–23 (35%)	40	38	25	49
24–35 (24%)	39	34	24	51
36–47 (13%)	35	33	22	49
49–59 (8%)	34	34	20	49
birth order				
1st (22%)	40	35	25	53
2nd (20%)	39	38	28	51
3rd (17%)	38	35	26	49
4th (14%)	38	35	26	48
5th (10%)	38	31	24	47
6th or >(16%)	36	31	22	46
stunted				
normal–mild (65%)	39	37	26	52
moderate (19%)	37	36	23	50
severe (16%)	35	37	23	41
wasted				
normal–mild (88%)	38	35	25	50
moderate (9%)	38	30	25	45
severe (3%)	40	30	23	44
*cluster*				
residence type				
urban (26%)	50	41	32	43
rural (74%)	34	33	22	35

### 3.1 Maternal effects

Wealth and education were largely and positively associated with health investment (table 2). Children of mothers with primary or secondary education or higher had higher odds of health investment than children of mothers without formal education and there was a high and positive increase in odds of investment by wealth quintile. In addition, there was a consistent increase in odds with maternal age across behaviours. For example, children of mothers aged 40–49 years had 40% higher odds of receiving ORS for diarrhoea than children of mothers aged 20–24 years (table 2). Polygynously married women had lower odds of providing bed net use and full immunization coverage but there was no difference compared with monogamously married women for fever treatment or ORS. Mothers who were severely wasted, compared to those only marginally wasted, had 13% and 19% lower odds, respectively, of providing fever treatment and ORS for their children.

**Table 2.** Logistic multilevel models predicting investment in child health (fever treatment, ORS, bed net use and immunization). *σ*^2*u*^ variance at country level and *σ*^2*e*^ at regional level (standard error in parenthesis). Distance is the mean number of women (%) within the sampling cluster who said distance to health centre was a barrier to seeking healthcare. OR, odds ratio; CI, credibility intervals; DIC, deviance information criterion.

	fever treatment	ORS	bed net use	immunization
	OR (95% CI)	OR (95% CI)	OR (95% CI)	OR (95% CI)
intercept	0.74 (0.53, 0.99)	0.40 (0.23, 0.79)	0.50 (0.26, 0.69)	1.69 (1.29, 2.27)
*maternal*				
age (years) (20–24)				
15–19	0.93 (0.82, 1.08)	0.99 (0.80, 1.19)	0.89 (0.79, 0.98)	0.89 (0.79, 1.00)
25–29	1.13 (1.02, 1.24)	1.08 (0.92, 1.26)	1.15 (1.06, 1.24)	1.12 (1.03, 1.21)
30–34	1.20 (1.06, 1.34)	1.09 (0.89, 1.32)	1.17 (1.06, 1.28)	1.21 (1.09, 1.34)
35–39	1.20 (1.04, 1.38)	1.19 (0.94, 1.49)	1.23 (1.09, 1.37)	1.20 (1.07, 1.35)
40–49	1.18 (1.00, 1.37)	1.40 (1.07, 1.85)	1.16 (1.01, 1.33)	1.22 (1.06, 1.39)
education (none)				
primary	1.37 (1.25, 1.49)	1.15 (1.01, 1.29)	1.21 (1.13, 1.30)	1.40 (1.31, 1.50)
secondary or >	1.55 (1.38, 1.75)	1.37 (1.14, 1.64)	1.52 (1.39, 1.66)	1.62 (1.48, 1.76)
wealth (poorest)				
poor	1.03 (0.93, 1.14)	1.24 (1.07, 1.45)	1.24 (1.14, 1.34)	1.07 (0.99, 1.16)
middle	1.13 (1.02, 1.25)	1.25 (1.08, 1.46)	1.34 (1.23, 1.46)	1.16 (1.08, 1.25)
rich	1.24 (1.11, 1.38)	1.37 (1.16, 1.62)	1.54 (1.41, 1.68)	1.26 (1.16, 1.37)
richest	1.61 (1.40, 1.82)	1.74 (1.41, 2.14)	2.56 (2.30, 2.85)	1.63 (1.48, 1.81)
marital status (monogamous)				
polygynous	0.99 (0.91, 1.07)	1.04 (0.91, 1.17)	0.82 (0.77, 0.87)	0.93 (0.87, 0.98)
not married	0.95 (0.85, 1.05)	0.84 (0.71, 0.98)	0.51 (0.46, 0.56)	0.89 (0.82, 0.96)
wasted (normal–mild)				
moderate	0.92 (0.86, 0.99)	0.98 (0.88, 1.09)	0.99 (0.94, 1.05)	1.00 (0.95, 1.06)
severe	0.87 (0.76, 0.98)	0.81 (0.66, 0.98)	0.92 (0.84, 1.02)	0.94 (0.86, 1.03)
*child*				
sex (boy)				
girl	0.88 (0.83, 0.93)	0.91 (0.82, 1.00)	1.01 (0.96, 1.06)	0.98 (0.93, 1.02)
age (months) (0–11)				
12–23	1.01 (0.93, 1.09)	1.40 (1.23, 1.58)	0.94 (0.88, 1.00)	ref
24–35	0.97 (0.89, 1.06)	1.18 (1.01, 1.36)	0.86 (0.80, 0.92)	1.09 (1.03, 1.16)
36–47	0.77 (0.68, 0.87)	1.14 (0.92, 1.38)	0.75 (0.69, 0.82)	0.95 (0.89, 1.02)
49–59	0.72 (0.62, 0.83)	1.12 (0.85, 1.45)	0.68 (0.61, 0.76)	0.88 (0.81, 0.96)
birth order (1st)				
2nd	0.95 (0.86, 1.05)	1.12 (0.96, 1.30)	1.08 (1.00, 1.17)	0.91 (0.83, 0.98)
3rd	0.93 (0.82, 1.04)	1.05 (0.87, 1.26)	0.93 (0.84, 1.02)	0.86 (0.78, 0.95)
4th	0.92 (0.80, 1.04)	1.10 (0.88, 1.35)	0.95 (0.85, 1.05)	0.81 (0.72, 0.91)
5th	0.92 (0.79, 1.07)	0.91 (0.70, 1.14)	0.86 (0.76, 0.96)	0.84 (0.74, 0.95)
6th or >	0.86 (0.73, 1.00)	0.94 (0.72, 1.19)	0.81 (0.71, 0.90)	0.80 (0.70, 0.91)
stunted (normal–mild)				
moderate	1.01 (0.93, 1.10)	1.01 (0.89, 1.14)	0.95 (0.89, 1.01)	1.00 (0.94, 1.07)
severe	1.03 (0.94, 1.13)	1.17 (1.03, 1.34)	0.90 (0.84, 0.96)	0.77 (0.72, 0.82)
wasted (normal–mild)				
moderate	1.14 (1.02, 1.26)	1.29 (1.10, 1.52)	0.94 (0.86, 1.03)	0.94 (0.86, 1.02)
severe	1.25 (1.04, 1.49)	1.34 (1.02, 1.72)	0.86 (0.73, 1.00)	0.88 (0.76, 1.02)
*environment*				
urban (rural)				
(cluster)	1.05 (0.95, 1.16)	1.09 (0.92, 1.27)	0.90 (0.83, 0.97)	0.95 (0.87, 1.02)
distance barrier				
(cluster)	0.50 (0.44, 0.56)	0.62 (0.51, 0.74)	0.75 (0.68, 0.83)	0.51 (0.46, 0.56)
under-5 mortality				
(region)	1.00 (1.00, 1.00)	1.00 (1.00, 1.00)	1.00 (0.99, 1.00)	1.00 (0.99, 1.00)
country				
*σ*^2*u*^	0.232 (0.100)	1.667 (0.676)	1.038 (0.563)	0.642 (0.267)
region				
*σ*^2*e*^	0.076 (0.017)	0.105 (0.031)	0.734 (0.114)	0.223 (0.035)
DIC	23 407.7	10 333.8	40 681.5	38 685.2

### 3.2 Child effects

Girls had lower odds of receiving both prompt fever/cough treatment and ORS than boys (12% and 9% lower odds, respectively), but there was no difference in investment for bed net use or full immunization coverage (table 2). There was a strong negative association between birth order and immunization (those with 5 or more older siblings had 20% lower odds of full immunization coverage) with similar trends for bed net use, fever treatment and ORS. The association between child health status and investment varied across health-seeking behaviours: for fever treatment and oral rehydration, wasted children had higher odds of treatment than normal weight children. However, for the preventative measures (bed net use and immunization), the relationship was the reverse; stunted and wasted children had lower odds of health investment (table 2). Both the maternal and the child effects changed in the full model when all other factors were included. Most notably, effects of maternal age were weaker or non-existent in the model with maternal and environmental variables only, but appeared when child factors were added to the model (see the electronic supplementary material, tables S2–S5).

## 4. Discussion

We have used DHS data to examine four different types of parental health-seeking behaviours with large impacts on child health. The results from this broad data resource show that health-seeking varies in a patterned fashion with several maternal and child characteristics, over and above strong positive effects of wealth and maternal education. We find evidence that these maternal and child effects are to a large extent independent of one another, and that not controlling for maternal and child factors simultaneously can result in misleading conclusions being drawn, for instance in the case of maternal age and birth order, discussed below. Sometimes a parental investment approach generates contradicting hypotheses for the influence of key maternal and child characteristics. Below we discuss our main findings and how they fit with different parental investment predictions, and speculate on some potential mechanisms.

### 4.1 Maternal age

Maternal age was a positive predictor of all the four health-seeking behaviours examined. Previous public health studies have found inconclusive results with respect to maternal age: that older mothers are less [20] or more [21,22] likely to invest in child health through diarrhoea treatment and immunization. However, these studies are often based on crude age categories (e.g. younger or older than 30 years) and often do not control for family size. It is a longstanding prediction from life-history theory that when reproductive value decreases with age, investment should increase with age [23]. In other words, because older mothers are less likely to have additional children in the future, they should be more likely to invest in the children they have (i.e. favour current over future reproduction). This is a simple life-history prediction that dates back to Williams [23] but one where evidence in the previous literature has not been conclusive [24,25]. McNamara *et al*. [26] used a more complex life-history model and predicted that if somatic condition deteriorates faster when the reproductive rate is high, older mothers should allocate less, not more, energy to reproduction. In line with this, it would be plausible to hypothesize that older women might comply less with health advice and favour traditional over newer advice on how to cure children. However, we find no evidence that older mothers invest less, so our results support the more traditional formulation that older mothers generally invest more in offspring.

### 4.2 Birth order

We found evidence of a disadvantage of higher birth order (i.e. being a later-born child) with respect to health investment. Competition in larger families was only significantly associated with lower investment for children with at least four older siblings, but consistent for all health behaviours. All else equal, larger family sizes mean that parental resources are diluted and less investment is available for each individual child. First-born offspring benefit from a period of sole investment which, all else equal, might lead to them having higher reproductive value than subsequent siblings. Siblings with lower birth order have also survived longer and are closer to reproducing than their younger siblings. However, the family is a unit within which not only competition but also cooperation between siblings occurs. Advantages to having several older siblings have been demonstrated, for example in terms of higher survival chances [16] and better mental health [27]. Moreover, it has been suggested that because younger children have higher need for intense parental investment, in some circumstances one unit of energy might actually yield higher marginal returns for younger than for older offspring [9,28]. It is also possible that benefits of scale, in terms of higher knowledge or access (e.g. sleeping under a bed net) mean that the parent can improve or maintain good health of several offspring at a lower cost per offspring in larger families. Because we are interested in behaviours pertaining to a child under 5, we use only data on the mother’s youngest child and cannot tease apart family size and birth order. Our findings of a later-born disadvantage concur with evidence on birth order effects on immunization in the Arsi Oromo of Ethiopia and the Karo Batak of Indonesia [29,30], but are here also seen for curative care for a sick child.

The results of a disadvantage of a higher birth order are particularly interesting in the light of the effect of maternal age, which appears to impact health investment in the opposite direction; all else equal, higher birth order children are born to older mothers. Notably, the positive maternal age effect was only seen when child characteristics were accounted for, indicating that offspring of older mothers differ in ways that can obscure the maternal age effect. A closer examination of the factors that covary with maternal age or birth order might help explain some of the causal mechanisms involved. For example, Wander & Mattison [31] found that later-born children in Tanzania were less likely to be weaned early, but that this effect disappeared when birth weight was controlled for. The authors did not find any evidence that older mothers had higher birth weight babies in this population. Other factors, such as interbirth intervals, might correlate with maternal age and influence parental investment. Assuming that age at first birth is the same, an older woman with a given number of children would have longer interbirth intervals on average and fewer young children that require care than a younger woman with the same number of children. If instead age at first birth is higher for the older woman, factors associated with a longer pre-reproductive life might be responsible for higher investment when she does commence childbearing, but we are not able to explore that relationship with these data.

### 4.3 Child health

We found evidence that severely wasted children had higher odds of receiving curative but lower odds of receiving preventative health investment. Several public health studies find that children in generally good health are more likely to be taken to a clinic [32], and that undernourishment is associated with delayed care [33]. However, many of these studies rely on retrospective health assessment by the mother, which might constitute a bias for the decision to treat a child. Our models that operationalize health status as stunting and wasting are not subject to such bias. From an evolutionary point of view, it is not necessarily clear what type of preferential investment might produce the highest fitness returns. On one hand, if a child is likely to die regardless of the parent’s efforts, it would be an adaptive strategy to allocate resources to other children that are more likely to benefit from them. Indeed, it is not uncommon for mothers to shift resources between children based on the child’s health, from mild biases to severe neglect [34–36]. Conversely, if investment in a ‘needy’ child can render increased survival chances, investment might be favoured; it has been found that mothers sometimes respond to children who ‘fuss’ more by providing longer breastfeeding [37].

Ultimately, whether poor child health should increase or decrease investment is likely to depend on how severe the child morbidity is and the type of investment. These results suggested that severely wasted children had higher odds of curative, but lower odds of preventative health-seeking and that this difference was less stark for stunting. This unexpected pattern might be due to the fact that while wasting is a measure of acute malnourishment, stunting results from long-term malnourishment and is a chronic state that is more difficult for parents to recuperate with health-seeking behaviour. In this light, it is unsurprising that parents seem to invest more in preventative behaviours for children in good nutritional health who are more likely to survive and benefit from longer term investments. Mothers with well-nourished children may have lower rates of ‘future discounting’; it has been argued that devaluing the future and favouring short-term benefits has evolved as a response to the need to weigh the present more highly when mortality risks or other extrinsic risks are high [38]. On a proximate level, it is possible that the decision to treat a child in need might be subject to a different decision-making mechanism and could invoke a stronger emotional response than decisions regarding pre-empting illnesses for healthy offspring.

### 4.4 Maternal health

We found evidence that wasting in women was associated with lower odds especially for the curative health-seeking behaviours, even when wealth was controlled for. Mothers in poor health may face a greater need to allocate resources towards their own somatic maintenance, which decreases the level of energy available for child health. Effects of maternal health are likely to be particularly important in harsh environments where diverting resources from maternal health jeopardizes the mother’s own survival. The trade-off between reproduction and maternal longevity has been studied in various populations [39,40] but evidence of how maternal investment varies as a function of health is very scarce, and complicated by the fact that healthier mothers often differ in important ways from less healthy mothers, for example in terms of wealth, marital status, age and number of offspring, that can all influence investment behaviour. It is also possible that mothers in poor health face practical constraints in taking a child to the clinic or performing health-seeking behaviours that we have not been able to control for fully. Overall, if there are conflicts of interest between parent and offspring, mothers may value themselves over their offspring when under stress that threatens their own survival prospects.

### 4.5 Marital status

There was evidence that monogamously married women had higher odds of investment than non-married women in all health-seeking behaviours. All else equal, single mothers are disadvantaged in childrearing because of lack of paternal care. Father absence has been examined in a range of contexts but mostly in terms of negative effects on child outcomes rather than parental behaviour. It is possible that mothers experiencing single parenthood may suffer larger constraints to preventative health-seeking than to the curative behaviours due to reduced paternal investment in the family; or the dilution or the absence of paternal investment may reduce long-term maternal investment in the children whose fathers may not be heavily invested in them because they are prioritizing future relationships [36].

Polygynously married women had lower odds of preventative health-seeking behaviour, but did not differ compared with monogamous women in terms of curative treatment. The consequences of polygyny is a classic question within evolutionary anthropology and the root causes and magnitude of the health disadvantages of polygynous families have been widely debated [15,41,42]. Polygynously married women have to compete with co-wives for investment from their husbands, potentially lowering the amount of resources available for healthcare. However, the polygyny threshold model predicts that polygyny will only occur when the costs of sharing a husband are offset by equal or greater resource access than can be obtained via monogamous marriage [43]. If this ‘female choice’ perspective on polygyny is correct, there should be no or little negative impact of polygyny on child health. Lawson and colleagues [44] recently demonstrated that while there was an overall negative association between polygyny and child health (measured as wasting and stunting) in an aggregated sample of 56 Tanzanian villages, there was no evidence of harmful effects of polygyny *within* villages. This study highlights that relationships between polygyny and aggregated child outcome data can be vulnerable to issues of confounding. In the Tanzanian case, associations between polygyny and poor health at the village level are driven by the fact that polygyny is most common in relatively marginalized and ecologically vulnerable ethnic groups. In the light of these findings, we are hesitant to make strong inferences about the effect of polygyny based on DHS data that do not comprise detailed data at the local community level. It is also not clear why we find a disadvantage of polygyny in the preventative but not the curative behaviours. We encourage scholars with access to detailed local data, in addition to examining child outcomes, to also explore various investment behaviours of polygynous mothers in future studies.

### 4.6 Sex of the child

We found evidence that girls are disadvantaged in terms of the curative behaviours, but there was no support for a sex bias in the preventative behaviours. Sex-biased investment can arise if parents expect higher net fitness benefits from one sex than the other due to one sex being more costly to raise; a particular sex might be favoured if that sex is more important in helping with work on important resources (local resource enhancement), or one sex may compete with parents for local resources (local resource competition) [45,46]. Several health-seeking studies from SSA report no significant sex biases [3,47,48] and, overall, male biases are more common in India, China and other parts of Asia than in SSA [49]. However, Cronk [50] found that girls among the Mukogodo in Kenya were more likely to be taken to a clinic for treatment and more likely to be treated for minor illnesses than boys. Compared to behaviours that are responses to illness events, preventative behaviour such as the habitual use of a bed net might benefit several offspring at once and scheduled immunizations can be part of a healthcare campaign where parents might be encouraged to invest. All these factors might potentially explain why we found evidence of a bias in the curative but not in the preventative behaviours.

### 4.7 General discussion

We have attempted to provide a broad view of how key maternal and child effects are associated with health-seeking behaviour as a proxy for direct parental investment. Parental behaviour has not been studied to the same extent as child outcomes and data on health-seeking behaviour offer an opportunity for testing this. A particular child outcome might have been caused by preventative or curative behaviour, or a combination of both, over a long or short time frame, but it is often difficult to determine the underlying causes for health outcomes. Our results highlight the importance of comparing various kinds of health-seeking behaviours with different time horizons in order to achieve a greater understanding of maternal investment in child health. Dupas [51] discusses costs and benefits associated with curative and preventative health expenditure from an economic perspective and provides examples that illustrate that individuals appear to be more sensitive to a change in price for preventative care than for curative treatment. Remarkably, experimental evidence from Kenya shows that even when prices on antimalarial tablets for children increase by 250% (from 0.30 USD to 1.50 USD) there is no decrease in households that buy the treatment [52]. Dupas [51] argues that this could be because not tending to an illness could cause high and immediate suffering that foregoing preventative health-seeking does not. Another difference between preventative and curative health-seeking is that the former is often provided by public health campaigns where the reliability of services is often poor. Even when health services are free of charge they tend to be used more by wealthier households, which could be because constraints might arise from cost of travel and opportunity costs of lost wages [53]. Thus, it is possible that preventative health-seeking imposes a double risk for vulnerable mothers—both in terms of whether the disease will actually be a threat in the future, and in terms of whether treatment can be attained when it is sought. Our results that preventative but not curative care was lower among mothers with poor health status, polygynously married mothers and children with poor health might in part be explained by these facts.

All measures used here are what is sometimes called ‘base-level investments’, i.e. vital for survival and different from ‘surplus investments’ that are not essential for survival, such as education [54]. It is therefore interesting that we find evidence of effects of child and maternal characteristics in these relatively ‘cheap’ behaviours. When studying biases between siblings it is worth bearing in mind that the type of investment and whether it is sequential or not is likely to influence how parents resolve allocation problems between offspring (see [55] for discussion of educational investments). Direct comparisons of how mothers bias investment within sibships are not possible if children require different resources at different times of their development; breastfeeding only benefits children for a certain period, and only school-aged children can be bestowed with investment in formal education. The health-seeking behaviours deployed here are only measured for children under 5 years of age, but other data resources with data on treatment for illnesses for offspring of different ages within families would be informative.

Evolutionary anthropologists have argued that the level of parental investment should be higher in environments where extrinsic mortality risk, i.e. mortality risk above individual control, is low, as parents are better able to influence child outcomes in such environments [56]. That many extrinsic factors continue to cause child deaths in SSA could explain why levels of investment have not risen more rapidly even when access and knowledge exists. Oster [57] found that women were more likely to use malaria bed nets if other causes of mortality were low, but such contextual influences remain difficult to disentangle with cross-sectional data. Interestingly, there is ample evidence from randomized controlled trials on subsidies for bed nets and immunizations showing that, also in high mortality contexts, health-seeking behaviour for children can be changed with relatively low cash incentives [51,58]. This is promising and consistent with the view that mothers’ trade-offs between managing child health and demands such as feeding and clothing children are relaxed with such cash incentives.

There has been an increase in human behavioural ecological studies using large-scale health and demographic data and focal parental investment questions have potential to be advanced by further intermarriage with population health sciences [15,59,60]. A natural next step is to test whether health-seeking behaviours incur real fitness trade-offs, a question that necessitates longitudinal data. Questions regarding interactions with the local environment will be better addressed by small-scale data collection efforts that are committed to measuring local constraints across varying contexts (e.g. NGOs with a between village design). Future research based on small-scale studies might be necessary to see how multiple forms of health-seeking for children vary within mothers, and how they relate to other measures such as age at weaning and investment in education. An important benefit of cross-country data such as the DHSs is that they can lend validity to small studies where breadth has been traded for long-term depth. If patterns found here, for example that maternal age is a positive predictor of investment *behaviour*, are also found in smaller studies that measure health *outcomes*, this will underpin evidence from both types of studies. Large-scale data also have great potential to pick up general patterns and indicate new areas for research for projects of various scales. Our finding that preventative and curative behaviours might be driven by different factors could be incorporated into future research projects and shed light on mechanisms involved.

## Supplementary Material

The supplementary materials includes table on descriptive statistics of the sample (TABLE S1), and model building tables for the final models (TABLES S2–S5).
