# Supplementary material for: Parental investment in child health in sub-Saharan Africa: a cross-national study of health-seeking behaviour
Source: R Soc Open Sci. 2016 Feb 24;3(2):150460. doi: 10.1098/rsos.150460 (PMC4785970; doi:10.1098/rsos.150460)
Supplement: The supplementary materials includes table on descriptive statistics of the sample (TABLE S1), and model building tables for the final models (TABLES S2–S5). [file rsos150460supp1.docx]

| Countries | Bed net use % | Oral rehydration solution for children who had diarrhoea % | Full immunisation coverage  (children aged 12-59 months) % | Treatment or advice sought for fever/cough % | Year |
| --- | --- | --- | --- | --- | --- |
| Burkina Faso | 19 | 20 | 36 | 35 | 2003 |
| Congo (DR) | 27 | 32 | 26 | 40 | 2007 |
| Cameroon | 13 | 23 | 37 | 38 | 2004 |
| Ethiopia | 4 | 22 | 18 | 18 | 2005 |
| Ghana | 17 | 38 | 52 | 43 | 2003 |
| Guinea | 14 | 32 | 32 | 32 | 2005 |
| Liberia | N/A | 55 | 26 | 58 | 2006/7 |
| Lesotho | N/A | 36 | 48 | 50 | 2004/5 |
| Mali | 44 | 15 | 36 | 32 | 2006 |
| Malawi | 24 | 63 | 51 | 33 | 2004/5 |
| Niger | 21 | 20 | 25 | 47 | 2006 |
| Rwanda | 18 | 11 | 56 | 27 | 2005 |
| Sierra Leone | 35 | 68 | 30 | 45 | 2008 |
| Senegal | 19 | 15 | 42 | 39 | 2005 |
| Swaziland | 1 | 87 | 65 | 50 | 2006/7 |
| Zambia | 41 | 62 | 16 | 62 | 2007 |
| Zimbabwe | 33 | 6 | 41 | 26 | 2005/6 |
| **Total** | 25 | 35 | 37 | 39 |  |
| Table S1. Mean level of health-seeking behaviour by country. | | | | | |

Electronic Supplementary Material: Parental investment in child health in sub-Saharan Africa.

|  |  |  | **Model a** | **Model b** | **Model c** | **Model d** |
| --- | --- | --- | --- | --- | --- | --- |
|  |  |  | OR(95% CI) | OR(95% CI) | OR(95% CI) | OR(95% CI) |
|  | Intercept |  | 1.17(0.89,1.44) | 0.65(0.43,0.93) | 1.19(0.93,1.54) | 0.74(0.53,0.99) |
| Mother | Age (years)  (20-24) | 15-19 |  | 0.98(0.87,1.10) |  | 0.93(0.82,1.08) |
|  |  | 25-29 |  | 1.07(0.98,1.17) |  | 1.13(1.02,1.24) |
|  |  | 30-34 |  | 1.10(1.00,1.21) |  | 1.20(1.06,1.34) |
|  |  | 35-39 |  | 1.05(0.95,1.17) |  | 1.20(1.04,1.38) |
|  |  | 40-49 |  | 0.96(0.85,1.08) |  | 1.18(1.00,1.37) |
|  | Education  (none) | Primary |  | 1.37(1.25,1.49) |  | 1.37(1.25,1.49) |
|  |  | Secondary |  | 1.55(1.38,1.74) |  | 1.55(1.38,1.75) |
|  | Wealth  (poorest) | Poor |  | 1.03(0.93,1.13) |  | 1.03(0.93,1.14) |
|  |  | Middle |  | 1.12(1.01,1.24) |  | 1.13(1.02,1.25) |
|  |  | Rich |  | 1.23(1.10,1.37) |  | 1.24(1.11,1.38) |
|  |  | Richest |  | 1.57(1.38,1.80) |  | 1.61(1.40,1.82) |
|  | Marital status  (monogamous) | Polygynous |  | 0.99(0.92,1.07) |  | 0.99(0.91,1.07) |
|  |  | Not married |  | 0.94(0.85,1.04) |  | 0.95(0.85,1.05) |
|  | Health  (normal-mild) | Moderate |  | 0.93(0.86,1.00) |  | 0.92(0.86,0.99) |
|  |  | Severe |  | 0.88(0.78,1.00) |  | 0.87(0.76,0.98) |
| Child | Sex (boy) | Girl |  |  | 0.88(0.82,0.93) | 0.88(0.83,0.93) |
|  | Age (months)  (0-11) | 12-23 |  |  | 1.03(0.95,1.11) | 1.01(0.93,1.09) |
|  |  | 24-35 |  |  | 1.01(0.92,1.08) | 0.97(0.89,1.06) |
|  |  | 36-47 |  |  | 0.81(0.73,0.91) | 0.77(0.68,0.87) |
|  |  | 49-59 |  |  | 0.77(0.66,0.88) | 0.72(0.62,0.83) |
|  | Birth order  (1^st^) | 2^nd^ |  |  | 0.98(0.89,1.08) | 0.95(0.86,1.05) |
|  |  | 3^rd^ |  |  | 0.99(0.89,1.09) | 0.93(0.82,1.04) |
|  |  | 4^th^ |  |  | 0.98(0.88,1.10) | 0.92(0.80,1.04) |
|  |  | 5^th^ |  |  | 0.98(0.87,1.11) | 0.92(0.79,1.07) |
|  |  | 6^th^ or > |  |  | 0.91(0.81,1.01) | 0.86(0.73,1.00) |
|  | Stunted  (normal-mild) | Moderate |  |  | 0.97(0.90,1.05) | 1.01(0.93,1.10) |
|  |  | Severe |  |  | 0.97(0.89,1.06) | 1.03(0.94,1.13) |
|  | Wasted | Moderate |  |  | 1.10(0.98,1.22) | 1.14(1.02,1.26) |
|  | (normal-mild) | Severe |  |  | 1.18(0.97,1.40) | 1.25(1.04,1.49) |
| Environ-  ment | Urban (rural) | (cluster) | 1.33(1.22,1.45) | 1.05(0.95,1.16) | 1.35(1.24,1.47) | 1.05(0.95,1.16) |
|  | ‘Distance to care’ | (cluster) | 0.44(0.39,0.49) | 0.50(0.44,0.56) | 0.44(0.39,0.50) | 0.50(0.44,0.56) |
|  | Under-five mortality | (region) | 1.00(1.00,1.00) | 1.00(1.00,1.00) | 1.00(1.00,1.00) | 1.00(1.00,1.00) |
| Country | Variance (s.e.) |  | 0.223(0.094) | 0.236(0.103) | 0.216(0.092) | 0.232(0.100) |
| Region | Variance (s.e.) |  | 0.071(0.01) | 0.075(0.016) | 0.074(0.160) | 0.076(0.017) |
| DIC |  |  | 23604.7 | 23452.8 | 23564.5 | 23407.7 |
| **Table S2.** Multilevel logistic models for **fever treatment**. Model a, environmental controls, model b maternal characteristics + environmental controls, model c: child characteristics + environmental controls, model d: full model (maternal + child + environmental characteristics). | | | | | | |

|  |  | |  | | **Model a** | | **Model b** | | **Model c** | | **Model d** | |  |
| --- | --- | --- | --- | --- | --- | --- | --- | --- | --- | --- | --- | --- | --- |
|  |  | |  | | OR(95% CI) | | OR(95% CI) | | OR(95% CI) | | OR(95% CI) | |  |
|  | Intercept | |  | | 0.92(0.46,1.50) | | 0.47(0.26,0.87) | | 0.77(0.34,1.46) | | 0.40(0.23,0.79) | |  |
| Mother | Age (years)  (20-24) | | 15-19 | |  | | 0.93(0.77,1.12) | |  | | 0.99(0.80,1.19) | |  |
|  |  |  | 25-29 | |  | | 1.09(0.95,1.24) | |  | | 1.08(0.92,1.26) | |  |
|  |  | | 30-34 | |  | | 1.04(0.89,1.21) | |  | | 1.09(0.89,1.32) | |  |
|  |  | | 35-39 | |  | | 1.10(0.92,1.29) | |  | | 1.19(0.94,1.49) | |  |
|  |  | | 40-49 | |  | | 1.24(1.01,1.49) | |  | | 1.40(1.07,1.85) | |  |
|  | Education  (none) | | Primary | |  | | 1.15(1.00,1.29) | |  | | 1.15(1.01,1.29) | |  |
|  |  |  | Secondary | |  | | 1.34(1.12,1.60) | |  | | 1.37(1.14,1.64) | |  |
|  | Wealth  (poorest) | | Poor | |  | | 1.24(1.07,1.42) | |  | | 1.24(1.07,1.45) | |  |
|  |  |  | Middle | |  | | 1.25(1.08,1.45) | |  | | 1.25(1.08,1.46) | |  |
|  |  | | Rich | |  | | 1.37(1.16,1.59) | |  | | 1.37(1.16,1.62) | |  |
|  |  | | Richest | |  | | 1.71(1.41,2.07) | |  | | 1.74(1.41,2.14) | |  |
|  | Marital status  (monogamous) | | Polygynous | |  | | 1.04(0.92,1.17) | |  | | 1.04(0.91,1.17) | |  |
|  |  |  | Not married | |  | | 0.84(0.71,0.98) | |  | | 0.84(0.71,0.98) | |  |
|  | Health  (normal-mild) | | Moderate | |  | | 1.02(0.91,1.13) | |  | | 0.98(0.88,1.09) | |  |
|  |  |  | Severe | |  | | 0.86(0.71,1.03) | |  | | 0.81(0.66,0.98) | |  |
| Child | Sex (boy) | | Girl | |  | |  | | 0.91(0.82,1.00) | | 0.91(0.82,1.00) | |  |
|  | Age (months)  (0-11) | | 12-23 | |  | |  | | 1.42(1.27,1.60) | | 1.40(1.23,1.58) | |  |
|  |  |  | 24-35 | |  | |  | | 1.22(1.06,1.39) | | 1.18(1.01,1.36) | |  |
|  |  | | 36-47 | |  | |  | | 1.19(0.98,1.43) | | 1.14(0.92,1.38) | |  |
|  |  | | 49-59 | |  | |  | | 1.21(0.93,1.55) | | 1.12(0.85,1.45) | |  |
|  | Birth order  (1^st^) | | 2^nd^ | |  | |  | | 1.15(1.00,1.32) | | 1.12(0.96,1.30) | |  |
|  |  |  | 3^rd^ | |  | |  | | 1.10(0.95,1.27) | | 1.05(0.87,1.26) | |  |
|  |  | | 4^th^ | |  | |  | | 1.17(0.99,1.37) | | 1.10(0.88,1.35) | |  |
|  |  | | 5^th^ | |  | |  | | 0.99(0.83,1.20) | | 0.91(0.70,1.14) | |  |
|  |  | | 6^th^ or > | |  | |  | | 1.08(0.92,1.27) | | 0.94(0.72,1.19) | |  |
|  | Stunted  (normal-mild) | | Moderate | |  | |  | | 0.98(0.87,1.11) | | 1.01(0.89,1.14) | |  |
|  |  |  | Severe | |  | |  | | 1.12(0.87,1.28) | | 1.17(1.03,1.34) | |  |
|  | Wasted | | Moderate | |  | |  | | 1.24(1.05,1.45) | | 1.29(1.10,1.52) | |  |
|  | (normal-mild) | | Severe | |  | |  | | 1.25(0.97,1.60) | | 1.34(1.02,1.72) | |  |
| Environ-  ment | Urban (rural) | | (cluster) | | 1.35(1.17,1.54) | | 1.10(0.94,1.29) | | 1.35(1.17,1.55) | | 1.09(0.92,1.27) | |  |
|  | ‘Distance to care’ | | (cluster) | | 0.56(0.46,0.68) | | 0.63(0.52,0.76) | | 0.56(0.46,0.67) | | 0.62(0.51,0.74) | |  |
|  | Under-five mortality | | (region) | | 1.00(1.00,1.00) | | 1.00(1.00,1.00) | | 1.00(0.99,1.00) | | 1.00(1.00,1.00) | |  |
| Country | Variance (s.e.) | |  | | 1.582(0.673) | | 1.700(0.704) | | 1.624(0.660) | | 1.667(0.676) | |  |
| Region | Variance (s.e.) | |  | | 0.010(0.029) | | 0.102(0.030) | | 0.102(0.032) | | 0.105(0.031) | |  |
| DIC |  | |  | | 10405.9 | | 10374.0 | | 10370.8 | | 10333.8 | |  |
| **Table S3.** Multilevel logistic models for **ORS**. Model a, environmental controls, model b maternal characteristics + environmental controls, model c: child characteristics + environmental controls, model d: full model (maternal + child + environmental characteristics). | | | | | | | | | | | | |  |
|  | | | | | | | | | | | | |  |
|  | |  | |  | | **Model a** | | **Model b** | | **Model c** | | **Model d** | |
|  | |  | |  | | OR(95% CI) | | OR(95% CI) | | OR(95% CI) | | OR(95% CI) | |
|  | | Intercept | |  | | 0.32(0.20.0.54) | | 0.23(0.13,0.40) | | 0.46(0.27,0.74) | | 0.50(0.26,0.69) | |
| Mother | | Age (years)  (20-24) | | 15-19 | |  | | 0.92(0.83,1.02) | |  | | 0.89(0.79,0.98) | |
|  |  |  |  | 25-29 | |  | | 1.08(1.01,1.16) | |  | | 1.15(1.06,1.24) | |
|  | |  | | 30-34 | |  | | 1.03(0.95,1.11) | |  | | 1.17(1.06,1.28) | |
|  | |  | | 35-39 | |  | | 1.00(0.92,1.09) | |  | | 1.23(1.09,1.37) | |
|  | |  | | 40-49 | |  | | 0.87(0.79,0.95) | |  | | 1.16(1.01,1.33) | |
|  | | Education  (none) | | Primary | |  | | 1.21(1.13,1.31) | |  | | 1.21(1.13,1.30) | |
|  | |  |  | Secondary | |  | | 1.55(1.43,1.70) | |  | | 1.52(1.39,1.66) | |
|  | | Wealth  (poorest) | | Poor | |  | | 1.25(1.15,1.35) | |  | | 1.24(1.14,1.34) | |
|  | |  |  | Middle | |  | | 1.34(1.24,1.45) | |  | | 1.34(1.23,1.46) | |
|  | |  | | Rich | |  | | 1.53(1.41,1.67) | |  | | 1.54(1.41,1.68) | |
|  | |  | | Richest | |  | | 2.56(2.29,2.85) | |  | | 2.56(2.30,2.85) | |
|  | | Marital status  (monogamous) | | Polygynous | |  | | 0.82(0.77,0.87) | |  | | 0.82(0.77,0.87) | |
|  | |  |  | Not married | |  | | 0.49(0.45,0.54) | |  | | 0.51(0.46,0.56) | |
|  | | Health  (normal-mild) | | Moderate | |  | | 0.99(0.94,1.04) | |  | | 0.99(0.94,1.05) | |
|  | |  |  | Severe | |  | | 0.91(0.84,1.00) | |  | | 0.92(0.84,1.02) | |
| Child | | Sex (boy) | | Girl | |  | |  | | 1.00(0.96,1.05) | | 1.01(0.96,1.06) | |
|  | | Age (months)  (0-11) | | 12-23 | |  | |  | | 0.97(0.91,1.03) | | 0.94(0.88,1.00) | |
|  | |  |  | 24-35 | |  | |  | | 0.89(0.83,0.95) | | 0.86(0.80,0.92) | |
|  | |  | | 36-47 | |  | |  | | 0.78(0.72,0.86) | | 0.75(0.69,0.82) | |
|  | |  | | 49-59 | |  | |  | | 0.71(0.64,0.79) | | 0.68(0.61,0.76) | |
|  | | Birth order  (1^st^) | | 2^nd^ | |  | |  | | 1.19(1.10,1.27) | | 1.08(1.00,1.17) | |
|  | |  |  | 3^rd^ | |  | |  | | 1.05(0.97,1.13) | | 0.93(0.84,1.02) | |
|  | |  | | 4^th^ | |  | |  | | 1.08(1.00,1.17) | | 0.95(0.85,1.05) | |
|  | |  | | 5^th^ | |  | |  | | 0.97(0.89,1.06) | | 0.86(0.76,0.96) | |
|  | |  | | 6^th^ or > | |  | |  | | 0.91(0.84,0.98) | | 0.81(0.71,0.90) | |
|  | | Stunted  (normal-mild) | | Moderate | |  | |  | | 0.90(0.84,0.96) | | 0.95(0.89,1.01) | |
|  | |  |  | Severe | |  | |  | | 0.83(0.78,0.90) | | 0.90(0.84,0.96) | |
|  | | Wasted | | Moderate | |  | |  | | 0.91(0.84,0.99) | | 0.94(0.86,1.03) | |
|  | | (normal-mild) | | Severe | |  | |  | | 0.81(0.70,0.94) | | 0.86(0.73,1.00) | |
| Environ-  ment | | Urban (rural) | | (cluster) | | 1.37(1.28.1.47) | | 0.91(0.84,0.98) | | 1.34(1.25,1.44) | | 0.90(0.83,0.97) | |
|  |  | ‘Distance to care’ | | (cluster) | | 0.64(0.58.0.71) | | 0.76(0.68,0.83) | | 0.64(0.58,0.70) | | 0.75(0.68,0.83) | |
|  | | Under-five mortality | | (region) | | 1.00(0.99.1.00) | | 1.00(0.99,1.00) | | 1.00(0.99,1.00) | | 1.00(0.99,1.00) | |
| Country | | Variance (s.e.) | |  | | 0.860(0.324) | | 0.847(0.459) | | 0.901(0.474) | | 1.038(0.563) | |
| Region | | Variance (s.e.) | |  | | 0.609(0.090) | | 0.681(0.104) | | 0.627(0.093) | | 0.734(0.114) | |
| DIC | |  | |  | | 41590.0 | | 40787.8 | | 41437.7 | | 40681.5 | |
| **Table S4.** Multilevel logistic models for **bed net use**. Model a, environmental controls, model b maternal characteristics + environmental controls, model c: child characteristics + environmental controls, model d: full model (maternal + child + environmental characteristics). | | | | | | | | | | | | | |
|  |  | |  | | **Model a** | | **Model b** | | **Model c** | | **Model d** | |  |
|  |  | |  | | OR(95% CI) | | OR(95% CI) | | OR(95% CI) | | OR(95% CI) | |  |
|  | Intercept | |  | | 2.77(1.95,3.72) | | 1.56(0.79,2.40) | | 2.42(1.51,3.84) | | 1.69(1.29,2.27) | |  |
| Mother | Age (years)  (20-24) | | 15-19 | |  | | 0.93(0.83,1.04) | |  | | 0.89(0.79,1.00) | |  |
|  |  |  | 25-29 | |  | | 1.04(0.97,1.12) | |  | | 1.12(1.03,1.21) | |  |
|  |  | | 30-34 | |  | | 1.08(1.00,1.17) | |  | | 1.21(1.09,1.34) | |  |
|  |  | | 35-39 | |  | | 1.04(0.95,1.13) | |  | | 1.20(1.07,1.35) | |  |
|  |  | | 40-49 | |  | | 1.02(0.94,1.11) | |  | | 1.22(1.06,1.39) | |  |
|  | Education  (none) | | Primary | |  | | 1.42(1.32,1.51) | |  | | 1.40(1.31,1.50) | |  |
|  |  |  | Secondary | |  | | 1.67(1.52,1.82) | |  | | 1.62(1.48,1.76) | |  |
|  | Wealth  (poorest) | | Poor | |  | | 1.08(1.00,1.17) | |  | | 1.07(0.99,1.16) | |  |
|  |  |  | Middle | |  | | 1.17(1.09,1.27) | |  | | 1.16(1.08,1.25) | |  |
|  |  | | Rich | |  | | 1.28(1.17,1.40) | |  | | 1.26(1.16,1.37) | |  |
|  |  | | Richest | |  | | 1.68(1.50,1.87) | |  | | 1.63(1.48,1.81) | |  |
|  | Marital status  (monogamous) | | Polygynous | |  | | 0.92(0.87,0.98) | |  | | 0.93(0.87,0.98) | |  |
|  |  |  | Not married | |  | | 0.90(0.83,0.97) | |  | | 0.89(0.82,0.96) | |  |
|  | Health  (normal-mild) | | Moderate | |  | | 1.00(0.94,1.05) | |  | | 1.00(0.95,1.06) | |  |
|  |  |  | Severe | |  | | 0.93(0.85,1.02) | |  | | 0.94(0.86,1.03) | |  |
| Child | Sex (boy) | | Girl | |  | |  | | 0.98(0.93,1.03) | | 0.98(0.93,1.02) | |  |
|  | Age (months)  (0-11) | | 12-23 | |  | |  | | Ref | | Ref | |  |
|  |  |  | 24-35 | |  | |  | | 1.11(1.05,1.18) | | 1.09(1.03,1.16) | |  |
|  |  | | 36-47 | |  | |  | | 0.99(0.92,1.06) | | 0.95(0.89,1.02) | |  |
|  |  | | 49-59 | |  | |  | | 0.93(0.85,1.00) | | 0.88(0.81,0.96) | |  |
|  | Birth order  (1^st^) | | 2^nd^ | |  | |  | | 0.96(0.89,1.03) | | 0.91(0.83,0.98) | |  |
|  |  |  | 3^rd^ | |  | |  | | 0.94(0.87,1.01) | | 0.86(0.78,0.95) | |  |
|  |  | | 4^th^ | |  | |  | | 0.89(0.82,0.96) | | 0.81(0.72,0.91) | |  |
|  |  | | 5^th^ | |  | |  | | 0.91(0.83,1.00) | | 0.84(0.74,0.95) | |  |
|  |  | | 6^th^ or > | |  | |  | | 0.87(0.80,0.94) | | 0.80(0.70,0.91) | |  |
|  | Stunted  (normal-mild) | | Moderate | |  | |  | | 0.96(0.90,1.02) | | 1.00(0.94,1.07) | |  |
|  |  |  | Severe | |  | |  | | 0.73(0.68,0.78) | | 0.77(0.72,0.82) | |  |
|  | Wasted | | Moderate | |  | |  | | 0.92(0.84,1.00) | | 0.94(0.86,1.02) | |  |
|  | (normal-mild) | | Severe | |  | |  | | 0.85(0.73,0.98) | | 0.88(0.76,1.02) | |  |
| Environ-  ment | Urban (rural) | | (cluster) | | 1.25(1.17,1.34) | | 0.95(0.89,1.04) | | 1.21(1.13,1.30) | | 0.95(0.87,1.02) | |  |
|  | ‘Distance to care’ | | (cluster) | | 0.44(0.40,0.49) | | 0.51(0.46,0.56) | | 0.45(0.41,0.50) | | 0.51(0.46,0.56) | |  |
|  | Under-five mortality | | (region) | | 1.00(0.99,1.00) | | 1.00(0.99,1.00) | | 1.00(0.99,1.00) | | 1.00(0.99,1.00) | |  |
| Country | Variance (s.e.) | |  | | 0.638(0.266) | | 0.681(0.296) | | 0.660(0.275) | | 0.642(0.267) | |  |
| Region | Variance (s.e.) | |  | | 0.220(0.034) | | 0.220(0.034) | | 0.220(0.034) | | 0.223(0.035) | |  |
| DIC |  | |  | | 39091.0 | | 38788.4 | | 38973.3 | | 38685.2 | |  |
| **Table S5.** Multilevel logistic models for **immunisation**. Model a, environmental controls, model b maternal characteristics + environmental controls, model c: child characteristics + environmental controls, model d: full model (maternal + child + environmental characteristics). | | | | | | | | | | | | |  |
